# Development of an LC-MS Targeted Metabolomics Methodology to Study Proline Metabolism in Mammalian Cell Cultures

**DOI:** 10.3390/molecules25204639

**Published:** 2020-10-12

**Authors:** Agnieszka Klupczynska, Magdalena Misiura, Wojciech Miltyk, Ilona Oscilowska, Jerzy Palka, Zenon J. Kokot, Jan Matysiak

**Affiliations:** 1 Department of Inorganic and Analytical Chemistry, Poznan University of Medical Sciences, 60-780 Poznan, Poland; jmatysiak@ump.edu.pl; 2 Department of Analysis and Bioanalysis of Medicines, Medical University of Bialystok, 15-222 Bialystok, Poland; magdalena.misiura@umb.edu.pl (M.M.); wojciech.miltyk@umb.edu.pl (W.M.); 3 Department of Medicinal Chemistry, Medical University of Bialystok, 15-222 Bialystok, Poland; ilona.zareba@gmail.com (I.O.); pal@umb.edu.pl (J.P.); 4 Faculty of Health Sciences, State University of Applied Sciences in Kalisz, 62-800 Kalisz, Poland; zkokot@ump.edu.pl

**Keywords:** metabolomics, cell culture, proline, amino acids, liquid chromatography-mass spectrometry

## Abstract

A growing interest in metabolomics studies of cultured cells requires development not only untargeted methods capable of fingerprinting the complete metabolite profile but also targeted methods enabling the precise and accurate determination of a selected group of metabolites. Proline metabolism affects many crucial processes at the cellular level, including collagen biosynthesis, redox balance, energetic processes as well as intracellular signaling. The study aimed to develop a robust and easy-to-use targeted metabolomics method for the determination of the intracellular level of proline and the other two amino acids closely related to proline metabolism: glutamic acid and arginine. The method employs hydrophilic interaction liquid chromatography followed by high-resolution, accurate-mass mass spectrometry for reliable detection and quantification of the target metabolites in cell lysates. The sample preparation consisted of quenching by the addition of ice-cold methanol and subsequent cell scraping into a quenching solution. The method validation showed acceptable linearity (r > 0.995), precision (%RSD < 15%), and accuracy (88.5–108.5%). Pilot research using HaCaT spontaneously immortalized human keratinocytes in a model for wound healing was performed, indicating the usefulness of the method in studies of disturbances in proline metabolism. The developed method addresses the need to determine the intracellular concentration of three key amino acids and can be used routinely in targeted mammalian cell culture metabolomics research.

## 1. Introduction

Culture cell metabolomics is a field of metabolomics that has the potential to reflect the phenotype of any cell and enhance our knowledge of cellular biochemistry, functions, and response mechanisms [1]. Cell metabolomics investigations aim to characterize profiles of small molecular weight compounds (typically less than 1500 Da) and usually focus on capturing changes associated with different signals or perturbations, e.g., gene mutations, pharmacological interventions, exposure to a toxin or stress agents. Moreover, the metabolomics studies employing cultured cells address key biological questions, such as metabolic flux in cells and tissues [2] and provide unique insights into metabolic phenomena in cells [3]. Therefore, metabolomics studies of cultured cells were demonstrated to be useful in many areas, including toxicology [4,5,6], pharmacology [7,8], cancer research [9,10,11,12], and biopharmaceutical production [13].

Metabolomics analysis of cell cultures offers many advantages compared with human and animal metabolomics research, where sometimes the researcher does not have strict control over the number of subjects and the type of samples available for a study. The use of established cell lines, animal or human-sourced, does not involve ethical concerns, which may restrict the number of samples or replicates [14]. Furthermore, the benefits of cell culture metabolomics include greater control of external variables, greater reproducibility, and easily interpreted results [14,15]. Variables such as age, sex, BMI, comorbidities, and other sources of inter-individual variability are not issues in culture cell analysis [15,16]. To conclude, the use of cultured cells, particularly mammalian cell lines, provides an opportunity to more carefully design intended research and achieve a stronger biological context of the acquired metabolomics data [1,14].

In cultured cell research, there is a need for the application of additional sample processing steps, such as cell metabolism quenching and metabolite extraction, compared to the use of biofluids that usually require only protein precipitation and dilution. The workflow in cell metabolomics encompasses a sequence of many steps, among which selection of cellular model, quenching, sample collection, metabolite extraction, normalization, and selection of analytical platform are the most important [14,15,17]. Heedful consideration of the methods employed for each step is required as it minimizes the risk of bias in the acquired data and allows for obtaining reliable results consistent with the purpose of the study.

The majority of the performed cell metabolomics studies were focused on an untargeted strategy (a global metabolite profiling), which aims to detect in a sample as a large number of metabolites as possible [18,19,20,21]. However, without knowing compounds of interest a priori, it is hard to develop the most suitable extraction protocols or optimize chromatographic separation. Consequently, there is a risk that some metabolites being critical for the interpretation of results co-elute with each other or are poorly separated. Thus, the metabolomics approach should depend on the study objective. When studying one specific metabolic pathway or a specific metabolite class, a targeted strategy is preferable [17,22]. Targeted metabolomics focuses on monitoring a predefined set of metabolites and permits the optimization of sample preparation protocol and analytical method parameters, offering a high level of precision and accuracy. Moreover, the targeted methods eliminate the need for sophisticated multi-step analysis of the huge amount of data, which is generated in an untargeted strategy to extract useful biological information.

**Figure 1:**
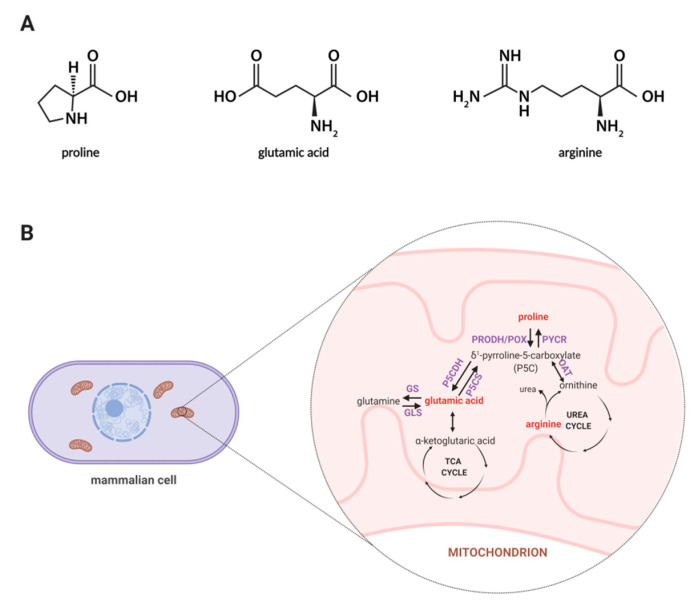
(**A**) Structures of proline, glutamic acid and arginine. (**B**) Proline metabolism in mammals. The proline metabolism involves the metabolism of other amino acids, such as ornithine, arginine, glutamic acid, and glutamine, and is related to the urea cycle and TCA cycle. Metabolites covered by the developed methodology are marked in red color. Abbreviations: GDH, glutamate dehydrogenase; GLS, glutaminase; GS, glutamine synthase; OAT, ornithine aminotransferase; PRODH/POX, proline dehydrogenase/oxidase; PYCR, P5C reductase; P5CDH, P5C dehydrogenase; P5CS, P5C synthase; TCA, tricarboxylic acid. Created with BioRender.com.

Among amino acids, proline seems to be unique, thus, exploring the role of proline in cellular metabolism is of an emerging interest as this amino acid affects many biological processes such as collagen biosynthesis, proline cycle, intracellular signaling, apoptosis, and autophagy [23,24]. Synthesis of proline involves the enzymatic conversion of pyrroline-5-carboxylic acid (P5C) derived from glutamate or ornithine [25]. In Figure 1A, the structures of proline, arginine, and glutamic acid are displayed, while Figure 1B represents a schematic illustration for the proline cycle and its relation to the tricarboxylic acid (TCA) and urea cycle where P5C acts as a mediator between these three cycles. Also, proline may originate from collagen degradation in which proline and its hydroxyl derivative (hydroxyproline) constitute about 25% of all amino acids [26]. An interesting area of active research involves the importance of proline in cell growth and proliferation. It is known that proline metabolism supports these processes through an enhanced synthesis of glutamine, glutamic acid, and nucleotides [27]. Thus, it is believed that proline-driven metabolic pathways may contribute to accelerated wound healing.

The study aimed to develop a robust and easy-to-use targeted metabolomics method for the determination of the intracellular level of proline and the other two amino acids closely related to proline metabolism: glutamic acid and arginine. Till now, only few methodologies have been developed to quantify amino acids in cell extracts [28,29,30,31,32]. Many of them employ a combination of a hydrophilic interaction liquid chromatography (HILIC) and a mass spectrometry (MS) detector, which indicates that it is a useful analytical platform for that purpose [30,31,32]. The above-cited methods rely on the application of a triple quadrupole mass analyzer operating in a multiple reaction monitoring mode. However, high-resolution mass spectrometers can also be successfully applied for metabolite quantification as they can effectively isolate analyte signal from the background noise and provide sensitive and reproducible measurements [33,34,35]. The developed method employs HILIC followed by high-resolution, accurate-mass MS for reliable detection and quantification of the target metabolites in cell lysates. The method was validated and then successfully applied to a pilot study of alterations of proline metabolism in spontaneously immortalized human keratinocytes HaCaT cell line. The results from both a series of validation tests and a pilot study on cell lines confirmed that the developed methodology could be used routinely in targeted mammalian cell culture metabolomics research.

## 2. Results and Discussion

### 2.1. Sample Preparation Method Development

**Figure 2:**
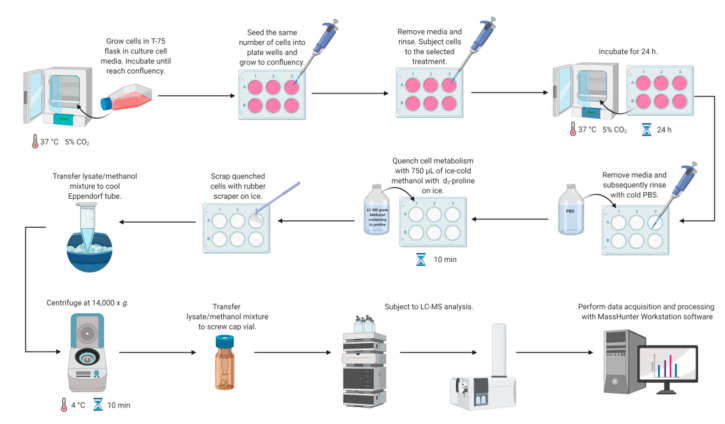
Workflow for LC-MS-based methodology for study proline metabolism in mammalian cell cultures. Created with BioRender.com.

Metabolomics studies in cultured cells require a strategy to collect samples in a manner to preserve metabolite concentration and then to extract cellular metabolites rapidly. Different quenching methods were described in the literature employing such solutions as ice-cold phosphate buffered-saline (PBS), ice-cold methanol, ice-cold 0.9% sodium chloride solution, or freezing in liquid nitrogen [14]. The developed method utilizes quenching by the addition of ice-cold methanol with subsequent cell scraping into a quenching solution. This method was shown to be beneficial compared to trypsinization, as it exhibits a higher extraction efficiency of metabolites [14,36,37,38]. Application of trypsin to detach adherent cells can alter the cellular metabolite levels due to its interaction with membrane proteins and can also cause a metabolite leakage through cell membranes [14,38,39]. Therefore, direct scraping of adherent cells was chosen as a method for harvesting (Figure 2).

Another critical step in sample preparation of cultured cells is metabolite extraction. In the developed method, extraction with ice-cold methanol was used. The selection of extraction solvent should be study-dependent and thus optimized depending on cell line and metabolites of interest. In untargeted metabolomics investigations, which aim to profile of a broad spectrum of metabolites, a dual-phase extraction using methanol, chloroform (or dichloromethane), and water is recommended. Another strategy is the application of methanol:water mixture (80:20, *v*/*v*) [14,40]. The extraction method used in our study was modeled on previous targeted and untargeted metabolomics studies, which utilized 100% methanol or 80% methanol [37,41]. According to the study of Bennett et al. [42], methanol extracts amino acids efficiently, and this solvent is preferred for research focused on this metabolite class. We performed a pilot study to compare the extraction efficiency of the target metabolites using two above-mentioned solvents: 100% methanol and 80% methanol. The conducted optimization study showed no significant differences in signals of analytes between extraction using pure methanol and methanol:water mixture (80:20, *v*/*v*). Similar extraction efficiency between these two solvents was also demonstrated by Dettmer et al. [37], who tested seven different extraction protocols in metabolomics research of adherently growing mammalian SW480 cells.

### 2.2. LC-MS Method Development

The determination of amino acids using chromatographic techniques is a challenging task. Due to a lack of volatility, amino acids require derivatization before gas chromatography [43,44]. Moreover, amino acids belong to polar metabolites, thus they usually exhibit low retention on a typical C18 column [45]. A traditional method for amino acid determination employs ion-exchange chromatography with post-column ninhydrin derivatization and UV detection [46]. A variety of liquid chromatography-based methods of amino acid determination have been developed, and most of them employ post- or pre-column derivatization [47,48,49]. Another alternative is the application of HILIC columns. HILIC constitutes a separation mode that employs a polar stationary phase and therefore, this technique is well suited for the analysis of low-molecular-weight polar molecules, such as organic acids or amino acids. HILIC methods require the high organic content of the mobile phase, which increases the ionization efficiency in an ion source and thus increases MS sensitivity. Due to the high compatibility with MS and the ability to retain polar metabolites, HILIC has grown in popularity in metabolomics studies, both untargeted and targeted [45,50,51]. In the developed method, the HILIC column was applied for the separation of underivatized amino acids, which allowed for the elimination of drawbacks related to chemical modifications of analytes.

**Figure 3:**
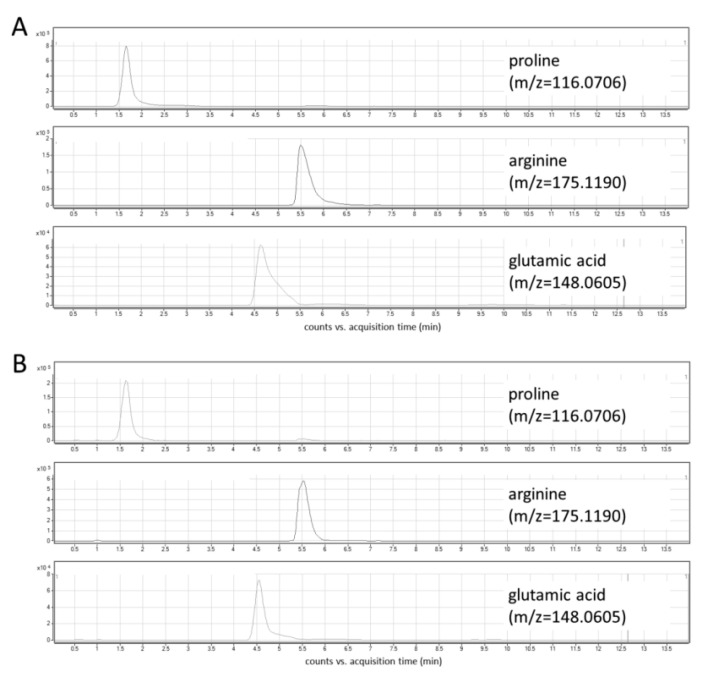
Extracted ion chromatograms of target metabolites: (**A**) standard solution; (**B**) cell lysate.

In this study, the optimization of chromatographic conditions in terms of mobile phase composition, gradient profile, flow rate, column temperature, and the injection volume was performed. Ammonium formate in two concentrations—5 mM and 10 mM—was tested as a mobile phase additive. Higher signal intensities and peak areas were obtained using a higher concentration of ammonium formate (Figure S1). Various column temperatures (30 °C and 45 °C, Figure S2), flow rates (0.6 mL/min and 0.8 mL/min), and injection volumes (2 μL and 5 μL) were also assessed. Three different gradient elution modes were tested, and finally, a gradient with increasing the water content up to 70% and the equilibration step lasting 6 min was selected. The optimized chromatographic parameters provided adequate separation of peaks of analytes at retention time ranges from 1.5 to 6.5 min (Figure 3A). The use of a reversed-phase (RP) column—Synergi Fusion RP (2 × 100 mm, 3 µm particle size, Phenomenex)—yielded unsatisfactory retention and separation of analytes as all of them eluted around 1.5 min.

The high-resolution mass spectrometer was used as a detector, which allows for the identification and quantification of target molecules with high sensitivity and specificity. Although triple-quadrupole mass spectrometry-based methods are commonly used for quantitation purposes, high-resolution systems, such as quadrupole-time of flight (Q-TOF) MS or quadrupole (Q)-Orbitrap MS, are also successfully used in metabolite determination [33,34,52]. They provide excellent mass accuracy (<5 ppm) and thus distinguish between analytes and some background interferences, which are not resolved with low-resolution mass spectrometers.

### 2.3. Method Validation

The developed methodology was validated in terms of specificity, carry-over, range, linearity, accuracy, precision, matrix effect, and stability. The successful validation allowed for the application of the method to the pilot study of cultured cells.

Under the optimized LC-MS conditions, no interferences at the retention times of the analytes were observed in cellular extracts (Figure 3B). In addition, three types of blanks were analyzed to exclude any potential solvent or system contribution: extraction blank (solvent-water treated in the same way as the real sample), solvent blank (methanol used for the metabolite extraction), and mobile phase blank. In all obtained chromatograms, no interfering signals were detected. An injection of a solvent blank following injection of the high concentration standard showed no carry-over at the retention times corresponding to analytes and IS.

All analytes showed acceptable linearity over the range of 1–50 µM (proline) or 2.5–50 µM (arginine, glutamic acid). The calculated correlation coefficients (r) for each amino acid were at least 0.995 and provided evidence of a good fit of the acquired data to the applied linear regression model.

Table 1 shows the intra- and inter-assay precision of the newly developed method calculated for peak areas and concentration values. The %RSD values obtained for concentrations ranged from 0.18% to 14.97% and from 0.66% to 14.27% for intra-day and inter-day precision, respectively. Moreover, as illustrated by the data contained in Table 1, the method gave excellent repeatability of the retention times of target metabolites. The percentage recoveries for the target amino acids are given in Table 2. The obtained values were in the range 88.5–108.5% indicating good accuracy of the method. The accuracy and precision of the method determined using spiking of a surrogate matrix (1 mg/mL bovine serum albumin (BSA) in PBS) with standard solutions of analytes were contained in Table S1. All calculated values fell within the acceptance criteria. The %RSD values obtained for concentrations ranged from 1.78% to 5.00% and from 2.75% to 10.83% for intra-day and inter-day precision, respectively. The calculated relative error values ranged from 1.46% to 13.62% and from 0.08% to 15.17% for intra-day and inter-day accuracy, respectively. The determined matrix effects ranged from 82.13% to 98.79% (Table 2).

The post-preparative stability of analytes, evaluated relative to fresh samples or fresh standard solutions, are summarized in Table 3. The data obtained show that the concentration of target metabolites did not change more than ± 15% after 24 h and 48 h storage in the autosampler. Therefore, the results indicate no substantial decline in response noted after the storage of solutions and samples at ambient temperature.

The performed validation proved that the developed method can be used as an alternative method for the determination of the intracellular concentration of proline and its’ related amino acids. The presented method has lower sensitivity compared with the existing LC-MS/MS methods, which employs a triple quadrupole mass spectrometer [31,32]. While the developed method does not provide the quantification limits of the most advanced triple-quadrupole systems, the sensitivity is more than sufficient for the study of intracellular concentration of proline, arginine, and glutamic acid, which was proved in the performed pilot study using HaCat cell line. Despite that limitation, the present method exhibits numerous advantages, such as a short time of analysis, simple sample preparation, high specificity, accuracy, and precision. The importance of proline in cell regulation [23,24,53] encourages the development of different analytical approaches that offer high throughput and absolute quantitation of metabolites of interest. The present method meets the conditions mentioned above. It constitutes a reliable tool in targeted studies of proline metabolism in cell cultures and can be expanded by adding new metabolites related to the proline metabolic axis, not only belonging to amino acids.

### 2.4. Application to Real Samples

As the proline cycle is reflected in many cellular processes, there is a possibility to apply this method for investigation of metabolic changes of proline and related metabolites under experimental conditions in vitro. The usefulness of the developed methods was proved in the study of proline, glutamic acid, and arginine in the cellular model for wound healing. We performed an analysis of target metabolites in the model for prolidase-promoted wound closure using the HaCaT cell line.

It is known that prolidase is a ligand of an epidermal growth factor receptor (EGFR) [54] promoting pro-growth and pro-proliferation signaling pathways. Since prolidase induces anabolic processes, we were interested in whether and how extracellular prolidase affects proline metabolism. In the prepared HaCaT cell extracts, we observed that each target amino acid occurred at a measurable level (above LLOQ). The obtained results showed that, when prolidase (c = 100 nM) was present in the cell culture medium, intracellular concentrations of proline, glutamic acid, and arginine were greatly increased. The results were normalized to protein concentration and they were calculated per µg protein. The proline concentration determined in prolidase-treated HaCaT cells was 6.31 ± 0.004 µM, whereas in control cells (with PBS as a vehicle) equaled 5.29 ± 0.02 µM. The level of glutamic acid rose from 26.32 ± 1.00 µM in control to 30.36 ± 0.10 µM in prolidase-treated HaCaT cells. Similarly, the concentration of arginine in control was 5.82 ± 0.11 µM while in treated cells increased to 8.72 ± 0.09 µM.

To sum up, the conducted pilot study proved that the developed method is adequate for monitoring the intracellular proline concentration in mammalian cell lines. The developed methodology applied for the study of HaCaT cells subjected to prolidase treatment under conditions of mechanical damage improved our knowledge about metabolic changes in intracellular cycles involved in EGFR-dependent promotion of wound healing. The final interpretation of the results is planned to be performed as it is beyond the study’s scope.

## 3. Materials and Methods

### 3.1. Reagents

Standards of analytes (L-proline, L-arginine, L-glutamic acid) and stable isotopically labeled internal standard (L-proline-d_3_, IS), were bought from Sigma Aldrich (St. Louis, MO, USA). LC-MS grade acetonitrile, formic acid, and ammonium formate were purchased from Sigma Aldrich (St. Louis, MO, USA). Deionized water (18.2 MΩ-cm resistivity at 25 °C) was obtained from the Milli-Q^®^ Advantage A10 water purification system (Merck Millipore, Darmstadt, Germany). The reference mass solution kit and tuning mix for calibrating the Q-TOF-MS were purchased from (Agilent Technologies, Santa Clara, CA, USA).

### 3.2. Preparation of Standard Solutions

Stock solutions (c = 0.5 M) of amino acids (analytes and IS) were prepared by dissolving the accurately weighed solids in deionized water. Working solutions of analytes (c = 5 mM; c = 0.5 mM; c = 0.05 mM) were made by mixing all analyte stock solutions together and then diluting the obtained mixture in methanol. Calibration standards were obtained by mixing an appropriate working solution with working IS solution and methanol. For each validation experiment, a new series of standard solutions were prepared.

### 3.3. Cell Culture

HaCaT spontaneously immortalized human keratinocytes were purchased from Cell Line Service GmbH (Eppelheim, Germany). HaCaT cells were cultured in DMEM cell culture medium (PanBiotech, Aidenbach, Germany) supplemented with 10% fetal bovine serum (FBS; Gibco, Waltham, MA, USA) and 1% Penicillin/Streptomycin (Gibco, Waltham, MA, USA). Cells were incubated at 37 °C in a humidified atmosphere of 5% CO_2_. The medium was replaced every 3 days until confluency. For LC-MS-based analysis of the selected amino acids, cells were seeded on 6-well plates at 2 × 10^5^ cells/well. At 80% of confluency, HaCaT cells were treated with 100 nM porcine prolidase (Sigma-Aldrich, Saint Louis, MO, USA) for 24 h. Cells subjected to PBS served as a control.

### 3.4. Quenching and Extraction

Simultaneous metabolism quenching and metabolite extraction employing ice-cold methanol was used for targeted metabolomics studies of adherent cell lines. The whole procedure was performed on ice. In the first step, the cell culture medium was aspirated and cells were washed quickly with 0.5 mL of PBS (pH 7.4) twice to remove any remaining culture medium. Then, 0.75 mL of 100% ice-cold methanol (−80 °C, LC-MS grade) containing proline-d3 (IS, c = 25 µM) was added to each well on a plate. The plates were incubated on ice for 10 min. Subsequently, cells were harvested using a rubber-tipped cell scraper. Cell lysate/methanol mixture was transferred to an Eppendorf tube, vortexed, and centrifuged (14,000× *g*; 4 °C; 10 min). The supernatant was transferred to an HPLC vial and subjected to LC-MS analysis. The concentrations of target metabolites were normalized to total protein content determined by BCA Protein Assay (Thermo Fisher Scientific, Waltham, MA, USA). Figure 2 shows all steps of the workflow in the LC-MS-based targeted analysis of proline, glutamic acid, and arginine in mammalian cell culture.

### 3.5. Instrumentation and Conditions

A 1260 Infinity high-performance liquid chromatograph (HPLC, Agilent Technologies, Santa Clara, CA, USA) hyphenated to a 6530 quadrupole-time of flight (Q-TOF) mass spectrometer equipped with a dual electrospray ionization (ESI) source (Agilent Technologies, Santa Clara, CA, USA) source was used in the study. Data acquisition and processing were performed with MassHunter Workstation software (Agilent Technologies, Santa Clara, CA, USA).

Chromatographic analyses were conducted using 5 μL of sample injected onto a Luna HILIC column (2 × 100 mm, 3 µm particle size, Phenomenex, Torrance, CA, USA). The mobile phase consisted of 10 mM ammonium formate with 0.1% formic acid in water (solvent A) and acetonitrile (solvent B). The optimized gradient elution profile was as follows: 0–2 min with 90% solvent B, 2–7 min linear from 90% to 30% solvent B, 7–7.5 min with 30% solvent B, 7.5–8 min from 30% to 90% solvent B, 8–14 with 90% solvent B. The flow rate was set at 0.6 mL/min and the column oven temperature was maintained at 30 °C. Before each injection, the needle was externally washed for 5 s with 50% water:50% isopropanol (1:1, *v*/*v*) using a flush port.

The mass spectrometer was operated in positive ion mode in full-scan MS mode, with nitrogen as the desolvation gas. The ESI source conditions were as follows: drying gas temperature, 325 °C; drying gas flow rate, 12.0 L/min; capillary voltage, 3000 V; skimmer voltage, 65 V; fragmentor voltage, 140 V and nebulizer gas pressure at 45 psi. Spectra were acquired in an *m*/*z* scan range of 50–1000 with an acquisition rate of 1 spec sec-1. During all analyses, two reference masses were used: *m*/*z* 121.0509 (protonated purine) and *m*/*z* 922.0098 [protonated hexakis (1*H*, 1*H*, 3*H*-tetrafluoropropoxy) phosphazine or HP-921]. The reference mass solution was continuously introduced into the dual ESI source, and the reference mass ions were measured to assure mass correction and mass accuracy performance verification. Samples were injected in random order. Each sample was injected in triplicate into the system.

### 3.6. Method Validation

Method linearity was evaluated by assaying seven non-zero calibration standards in triplicate. Due to the absence of an analyte-free matrix, calibration samples were prepared in methanol. They were prepared on the day of analysis and analyzed with the quality control (QC) samples as a single batch. The calibration curves were constructed using linear regression by plotting the peak area ratio of analyte to IS against the nominal concentration of analytes. The calibration standard with the lowest concentration level was regarded as the lower limit of quantification (LLOQ), having a signal to noise ratio (S/N) at least 10 and with accuracy within ± 20% of the nominal value, and signal variability represented as percent relative standard deviation (%RSD) not higher than 20%.

Carry-over was evaluated by injecting solvent blank sample (methanol) after the calibration standard with the highest concentration of analytes. An analyte signal in the blank sample after the analysis of a high concentration standard should not exceed 20% of the LLOQ, whereas an IS signal should not be greater than 5%.

Precision and recovery of the method were tested using QC samples at different concentration levels. QC samples were prepared by pooling supernatants obtained after metabolite extraction from cell lines. The intra-day precision involved five replicates of QC samples per each concentration level performed within a single run. To examine inter-day precision, QC samples were analyzed on five consecutive validation days. The precision was satisfactory when the calculated %RSD of replicates was not higher than 15%. Recovery was determined by spiking QC samples with different amounts of analyte standards and calculated as follows.

(1) $$\frac{\mathrm{spiked}\;\mathrm{sample}\;\mathrm{result}-\mathrm{unspiked}\;\mathrm{sample}\;\mathrm{result}}{\mathrm{known}\;\mathrm{spike}\;\mathrm{added}\;\mathrm{concentration}}\times 100\%$$

As the analytes were endogenous compounds, an additional set of QC samples, prepared by spiking of a surrogate matrix with standard solutions, was analyzed to evaluate method accuracy, precision, and matrix effect. BSA added to PBS is a frequently used surrogate matrix in the determination of endogenous metabolites, which simulates the pH, ionic strength, and protein content of the biological matrix [55,56,57]. We used 1 mg/mL BSA solution in PBS, which corresponds to the protein concentration in one well. The spiked surrogate matrix samples were prepared using one-step extraction with 0.75 mL of 100% ice-cold methanol, as described in Section 3.4. For accuracy determination, relative error (RE, bias) was calculated for each measurement according to the formula presented below.

(2) $$\frac{\mathrm{calculated}\;\mathrm{value}-\mathrm{nominal}\;\mathrm{value}}{\mathrm{nominal}\;\mathrm{value}}\times 100\%$$

Accuracy was found satisfactory when the RE was within ± 15%. The precision was satisfactory when the calculated %RSD of replicates was not higher than 15%. The matrix effect was evaluated by comparison of slopes of calibration curves prepared with and without the surrogate matrix.

Autosampler (post-preparative) stability was evaluated for both standard solutions and cell lysates after 24 h and 48 h storage in the autosampler (room temperature). Standard solutions at two concentration levels along with 2 QC samples were tested. The stability was calculated as (area ratio of a sample after storage/area ratio of a freshly prepared sample) × 100%.

## Figures and Tables

**Table 1:** Relative standard deviation (RSD) values in intra-day and inter-day precision tests of the developed method.

Analyte	Sample	Intra-Day Precision (n = 5)			Inter-Day Precision (n = 5)		
		RT ^1^ (min), %RSD ^2^	Peak Area, %RSD ^2^	Conc ^3^, %RSD ^2^	RT ^1^ (min), %RSD ^2^	Peak Area, %RSD ^2^	Conc ^3^, %RSD ^2^
Proline	QC1	0.04	2.95	0.18	0.92	10.53	0.66
	QC2	0.74	7.25	1.29	0.69	6.21	0.84
	QC3	1.06	7.13	1.71	0.78	6.07	1.24
Arginine	QC1	0.14	6.85	3.34	0.15	14.27	5.67
	QC2	0.18	1.59	12.41	0.23	4.98	8.46
	QC3	0.07	4.01	14.97	0.14	5.25	10.26
Glutamic acid	QC1	0.23	6.12	4.87	0.33	7.27	5.19
	QC2	0.45	8.04	0.92	0.41	5.21	3.61
	QC3	0.31	5.65	3.40	0.33	4.17	3.68

^1^ RT—retention time; ^2^ RSD—relative standard deviation; ^3^ Conc—concentration value.

**Table 2:** Mean percentage recoveries of the target metabolites and the determined matrix effect.

Analyte	Recovery, %			Matrix Effect ^1^ %
	QC1 (n = 3)	QC2 (n = 3)	QC3 (n = 3)	
Proline	103.76	99.96	105.84	98.79
Arginine	98.08	100.33	88.46	96.03
Glutamic acid	103.01	99.86	108.54	82.13

^1^ determined using a surrogate matrix (1 mg/mL BSA in PBS).

**Table 3:** Results of post-preparative stability tests of target metabolites.

Analyte	Sample	24 h Storage		48 h Storage	
		Peak Area, %	Conc ^1^, %	Peak Area, %	Conc ^1^, %
Proline	Standard solution	99.17	99.72	123.93	99.22
	Cell lysate	104.67	99.32	112.27	99.23
Arginine	Standard solution	101.25	102.97	118.05	93.24
	Cell lysate	98.12	91.63	100.35	86.64
Glutamic acid	Standard solution	94.66	95.12	118.03	93.97
	Cell lysate	105.47	100.03	111.28	98.27

^1^ Conc—concentration value.

## Supplementary Materials

The following are available online, Figure S1: Extracted ion chromatograms of target metabolites obtained using different mobile phase composition, Figure S2: Extracted ion chromatograms of target metabolites obtained using different column temperatures, Table S1: Intra-day and inter-day validation of the developed methodology performed using quality control (QC) samples at three different concentration levels. The QC samples were prepared by spiking of a surrogate matrix (1 mg/mL bovine serum albumin in PBS) with standard solutions of analytes.
