# Supplementary material for: Development of an LC-MS Targeted Metabolomics Methodology to Study Proline Metabolism in Mammalian Cell Cultures
Source: Molecules. 2020 Oct 12;25(20):4639. doi: 10.3390/molecules25204639 (PMC7587214; doi:10.3390/molecules25204639)
Supplement: Supplementary file 1 [file molecules-25-04639-s001.pdf]

Supplementary Materials

Development of an LC-MS targeted metabolomics methodology to study proline metabolism in mammalian cell cultures

Agnieszka Klupczynska [1,*,†], Magdalena Misiura [2,†], Wojciech Miltyk [2], Ilona Oscilowska [3], Jerzy Palka [3], Zenon J. Kokot [4] and Jan Matysiak [1]

[1] Department of Inorganic and Analytical Chemistry, Poznan University of Medical Sciences, 60 -780 Poznan, Poland
[2] Department of Analysis and Bioanalysis of Medicines, Medical University of Bialystok, 15-222 Bialystok, Poland
[3] Department of Medicinal Chemistry, Medical University of Bialystok, 15-222 Bialystok, Poland
[4] Faculty of Health Sciences, State University of Applied Sciences in Kalisz, 62-800 Kalisz, Poland

***** Correspondence: aklupczynska@ump.edu.pl
† Equal contribution

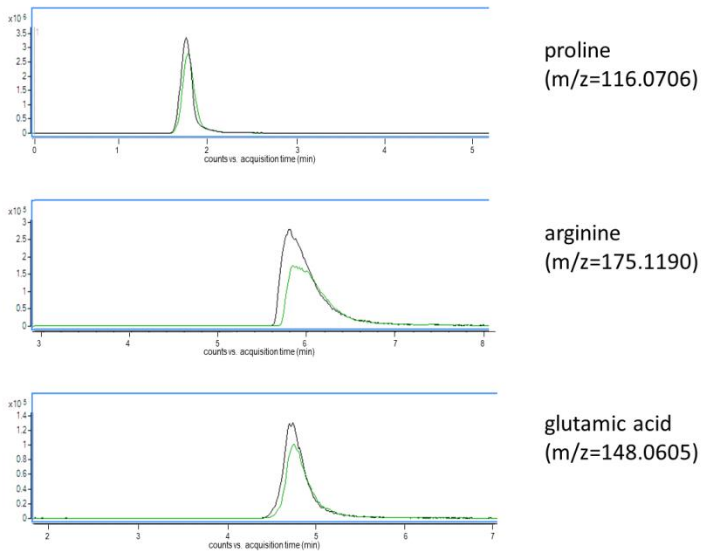

Figure S1. Extracted ion chromatograms of target metabolites obtained using different mobile phase composition. Black color – solvent A: 10 mM ammonium formate with 0.1% formic acid in water; solvent B: acetonitrile. Green color – solvent A: 5 mM ammonium formate with 0.1% formic acid in water; solvent B: acetonitrile.

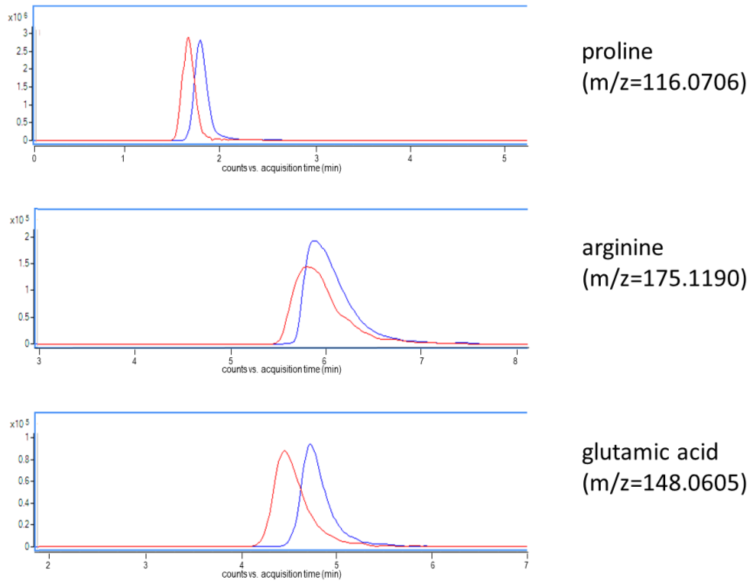

Figure S2. Extracted ion chromatograms of target metabolites obtained using different column temperatures. Blue color - 30°C. Red color - 45°C.

Table S1. Intra-day and inter-day validation of the developed methodology performed using quality control (QC) samples at three different concentration levels. The QC samples were prepared by spiking of a surrogate matrix (1 mg/mL bovine serum albumin in PBS) with standard solutions of analytes.

Analyte	Sample	Conc (μM)	Intra-day (n=5)				Inter-day (n=5)			
			RT [1] (min), %RSD [2]	Peak area, %RSD [2]	Conc, %RSD [2]	Relative error, %	RT [1] (min), %RSD [2]	Peak area, %RSD [2]	Conc, %RSD [2]	Relative error, %
Proline	QC low	5	0.57	2.46	1.90	-13.98	0.67	8.72	6.97	-10.40
	QC medium	15	0.39	3.34	3.23	-7.34	0.45	5.37	3.45	-5.47
	QC high	25	0.48	2.74	2.79	-2.67	0.42	4.20	2.75	-2.00
Arginine	QC low	5	0.27	4.12	5.00	13.62	0.23	6.60	8.20	15.17
	QC medium	15	0.07	1.39	1.78	3.92	0.12	10.25	8.12	6.87
	QC high	25	0.09	3.45	3.86	-3.54	0.12	12.13	10.83	0.08
Glutamic acid	QC low	5	0.46	3.52	2.64	1.46	0.43	7.41	7.35	-1.44
	QC medium	15	0.33	2.88	2.12	-11.77	0.35	8.28	8.86	-13.04
	QC high	25	0.18	3.37	3.38	-10.99	0.24	6.89	7.97	-13.88

[1] RT – retention time; [2] RSD – relative standard deviation; [3] Conc – concentration value
